# NLRP6-associated host microbiota composition impacts in the intestinal barrier to systemic dissemination of *Brucella abortus*

**DOI:** 10.1371/journal.pntd.0009171

**Published:** 2021-02-22

**Authors:** Marcella Rungue, Victor Melo, David Martins, Priscila C. Campos, Gabriela Leles, Izabela Galvão, Viviani Mendes, Mariana Aganetti, Ágatha Pedersen, Natan R. G. Assis, Raiany Santos, Geovanni D. Cassali, Ana Lúcia B. Godard, Flaviano S. Martins, Sergio C. Oliveira, Angélica T. Vieira

**Affiliations:** 1 Laboratory of Microbiota and Immunomodulation (LMI)- Department of Biochemistry and Immunology, Institute of Biological Sciences, Federal University of Minas Gerais, Belo Horizonte, Minas Gerais, Brazil; 2 Department of Microbiology, Institute of Biological Sciences, Federal University of Minas Gerais, Belo Horizonte, Minas Gerais, Brazil; 3 Department of General Biology, Institute of Biological Sciences, Federal University of Minas Gerais, Belo Horizonte, Minas Gerais, Brazil; 4 Department of General Pathology, Institute of Biological Sciences, Federal University of Minas Gerais, Belo Horizonte, Minas Gerais, Brazil; Oregon State University College of Veterinary Medicine, UNITED STATES

## Abstract

*Brucella abortus* is a Gram-negative bacterium responsible for a worldwide zoonotic infection—Brucellosis, which has been associated with high morbidity rate in humans and severe economic losses in infected livestock. The natural route of infection is through oral and nasal mucosa but the invasion process through host gut mucosa is yet to be understood. Studies have examined the role of NLRP6 (NOD-like receptor family pyrin domain-containing-6 protein) in gut homeostasis and defense against pathogens. Here, we investigated the impact of gut microbiota and NLRP6 in a murine model of *Ba* oral infection. Nlrp6^*-/-*^ and wild-type (WT) mice were infected by oral gavage with *Ba* and tissues samples were collected at different time points. Our results suggest that *Ba* oral infection leads to significant alterations in gut microbiota. Moreover, Nlrp6^-/-^ mice were more resistant to infection, with decreased CFU in the liver and reduction in gut permeability when compared to the control group. Fecal microbiota transplantation from WT and Nlrp6^*-/-*^ into germ-free mice reflected the gut permeability phenotype from the donors. Additionally, depletion of gut microbiota by broad-spectrum-antibiotic treatment prevented *Ba* replication in WT while favoring bacterial growth in Nlrp6^*-/-*^. Finally, we observed higher eosinophils in the gut and leukocytes in the blood of infected Nlrp6^*-/-*^ compared to WT-infected mice, which might be associated to the Nlrp6^*-/-*^ resistance phenotype. Altogether, these results indicated that gut microbiota composition is the major factor involved in the initial stages of pathogen host replication and partially also by the resistance phenotype observed in Nlrp6 -/- mice regulating host inflammation against *Ba* infection.

## Author summary

*Brucella abortus* (Ba) is an intracellular bacterium that causes zoonotic and clinical problems worldwide. Although the common route of infection is through oral and nasal, the mechanisms toward the gastrointestinal mucosa response is still unexplored. It is well known that microbiota promotes and maintains host intestinal homeostasis during bacterial infections. However, mechanisms by which the gut microbiota affects the Ba infection have not yet been demonstrated. Here, we provide significant insights into the relationship between gut microbiota and *B*. *abortus* oral infection and demonstrate the gut microbiota contribution to the gut permeability and dissemination of Ba. Furthermore, we investigated the participation of the gut microbiota from Nlrp6 deficient mice, on the gut permeability and Ba infection. Substantial experiments performed, mostly in vivo, showed that gut microbiota alterations promote gut barrier disruption, as indicated by increased gut permeability after Ba oral infection. Thus, our work highlights the role of gut mucosal environment through gut microbiota and Nlrp6 molecule involved in host innate immune responses to Ba infection.

## Introduction

Brucellosis is a zoonosis caused by the Gram-negative intracellular pathogen *Brucella spp*., with severe impacts on livestock productivity and human health worldwide [1]. Annually, it results in more than 500,000 human cases and losses of more than USD 600 million in Latin America due to animal injuries [2]. For this reason, the World Health Organization (WHO) classified it as one of the seven most neglected zoonotic diseases. In humans, the mortality is uncommon, and the disease is most often originated from an animal reservoir [3]; symptoms include intermittent fever, night sweats, anorexia, polyarthritis, meningitis and pneumonia. Infection primarily occurs following mucosal exposure to contaminated aerosols or through consumption of non-pasteurized dairy products from *Brucella*-infected animals, although it can also occur through direct contact with infected animals or tissues and fluids associated with abortion [4,5].

Rare studies use the more physiological intranasal or oral routes of *Brucella* infection; instead, the main experimental model for studying brucellosis in mice is intraperitoneal (i.p.) infection [4]. Therefore, expanding our knowledge on how the host immune responses limit the dissemination and bacterial invasion, especially in the gut mucosa, may allow us to develop novel therapeutic strategies. In this context, the intestinal epithelium constitutes the major barrier that separates the internal from the external environment, providing high resistance to the entry of pathogens [6,7]. Still, the intestine is composed by a vast and dense ecosystem of trillions of microorganisms ranging from bacteria, yeast, and fungi to viruses and protozoa. These innumerous microorganisms, called microbiota, in symbiosis, are crucial for the host’s health. Furthermore, the gut microbiota has shown to be crucial to host resistance against invading pathogens within the intestine, either directly (i.e. competition) or indirectly (i.e. enhancing host immunity) [8–11]. Notwithstanding, disruption of the microbiota composition has been associated with epithelial barrier breaks, leading to exacerbation of the inflammatory process, tissue damage and elevated risk of systemic invasion, thus elevating the possibility of bacterial translocation [10,12]. There are only a few articles studying the interaction between *Brucella*, the epithelial barrier and the immune response, in the gut mucosa [13,14] and, more precisely, the interaction between this pathogen and the host microbiota.

The gut microbiota not only promotes mucosal barrier function, but also enhances host immunity defense against enteric infections. In this context, NLRs (a family of nucleotide-binding domain, leucine-rich repeat-containing innate immune receptors) play an important role on the recognition of pathogens by epithelial cells [15]. NLRP6 (a member of the NLR family) is highly expressed in the small and large intestine and promotes the maturation and secretion of IL-18, a pro-inflammatory cytokine (through the NLRP6 inflammasome, which is believed to be responsible for repairing the epithelial barrier and cell proliferation and maturation [16–18]. NLRP6 inflammasome-induced IL-18 is modulated by microbial metabolites, and downstream IL-18 secretion induces an antimicrobial peptide program in intestinal epithelial cells that is critical to prevent dysbiosis [19].

In the present study, we elucidate the impact of different gut microbiota composition by using two different mouse strains from WT (C57/bl6) and NLRP6 (Nlrp6-/-) mice in a model of oral infection by *B*. *abortus*. Our results suggest that mice deficient in NLRP6 were more resistant to *B*. *abortus* infection since there was no disruption in intestinal barrier after infection. NLRP6 deficient mice present altered gut microbiota composition, which could impairs *B*. *abortus* epithelial invasion, as indicated by decreased bacterial burdens in the liver.

## Results

### Intestinal epithelial permeability is associated with *Brucella abortus* invasion after oral infection

**Fig 1:**
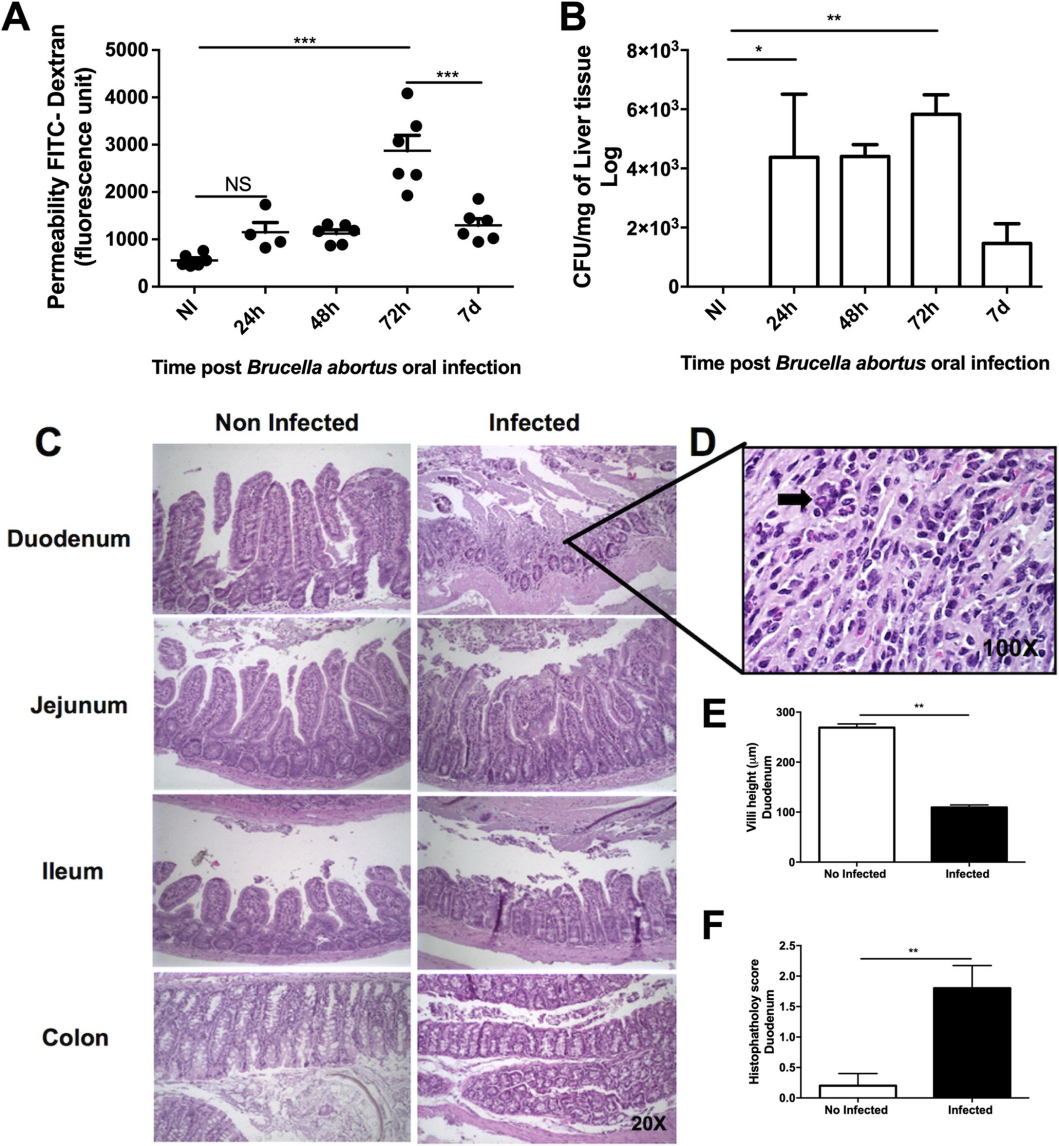
The pathophysiological effects of oral infection with *B*. *abortus*. **(a)** Increased intestinal permeability, especially after 3 days of infection. **(b)** Presence of viable bacterial load in the liver at all times of infection evaluated. **(c)** Representative photomicrography of the different segments of the small intestine and colon of infected (3 days post-infection) and non-infected animals. Tissue damage was observed mainly in the duodenum. (**d**) The arrow indicates eosinophilic infiltration. Tissue damage was observed mainly in the duodenum and collected for analysis of (E) villi height and (F) histopathology score. Results expressed as mean ± standard error of mean (n = 5–7). *p < 0.05; **p< 0.01; ***p < 0.001.

In order to evaluate the kinetics of *B*. *abortus* infection through the gastrointestinal tract, mice were infected with 1x10^9^ CFU of *B*. *abortus* by gavage. We understand that 1x10^9^ CFU per animal is a high inoculum, however, it was chosen due to the consistent colonization of the liver tissue and gut inflammation (as observed by neutrophils infiltration-MPO), compared to the highest (1x10^10^) and lowest (1x 10^8^) CFU dose (**S1 Fig**). The characterization of the kinetics of oral infection by *B*. *abortus* was analyzed by measuring the total intestinal permeability and the bacterial load in the liver of WT mice at different time points. We observed a significant increase in intestinal permeability 3 days post-infection [8], which was later reduced, after 7 days of infection (**Fig 1A**). It was also possible to recover viable bacteria in the liver of the infected animals at all time points assessed, decreasing after 7 days of infection (**Fig 1B**). Moreover, we characterized the tissue damage after infection with *B*. *abortus* using histopathologic analysis of fragments of the duodenum, jejunum and colon. The main alterations related to the breakdown of the intestinal epithelial barrier were observed in the duodenum, which showed flattening of the villi (**Fig 1E**) and inflammatory infiltration as quantified by the histological score (**Fig 1F**).

### NLRP6 inflammasome deficiency confers host intestinal resistance to *B*. *abortus* when the bacteria were administered orally but not intraperitoneally

**Fig 2:**
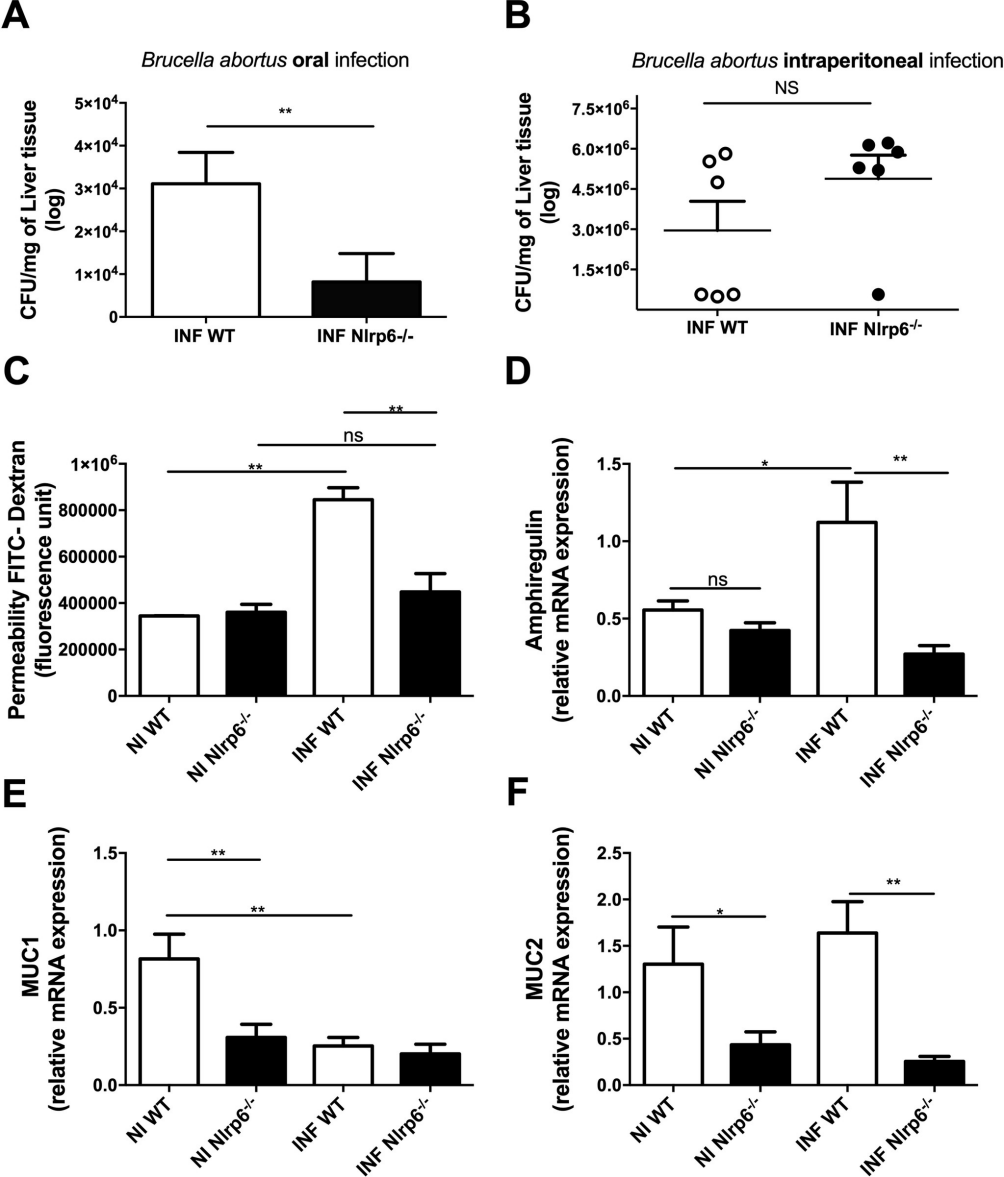
Nlrp6^-/-^ were more resistant to *Brucella abortus* oral infection than wild type mice. WT and Nlrp6^-/-^ mice were infected by both oral and intraperitoneal routes in two independent experiments. Euthanasia was performed after 3 days of infection **(a)** Increased a load of viable bacteria in the liver of WT animals after oral infection. **(b)** Equal viable bacterial load in the liver of WT and Nlrp6^-/-^ animals after systemic infection. **(c)** No alterations in intestinal permeability in infected Nlrp6^-/-^ animals. **(d)** Increased expression of mRNA to Amphiregulin in infected WT animals. **(e)** Nlrp6^-/-^ animals naturally have reduced expression of mRNA to MUC1. WT animals presented a reduction of MUC1 after infection. **(f)** There is no change in expression from mRNA to MUC2 in uninfected and infected animals. Times of 0h (non-infected animals) and 3 days were compared. Results expressed as mean ± standard error of mean (n = 5–7). *p < 0.05; **p < 0.01; ***p < 0.001.

**Fig 3:**
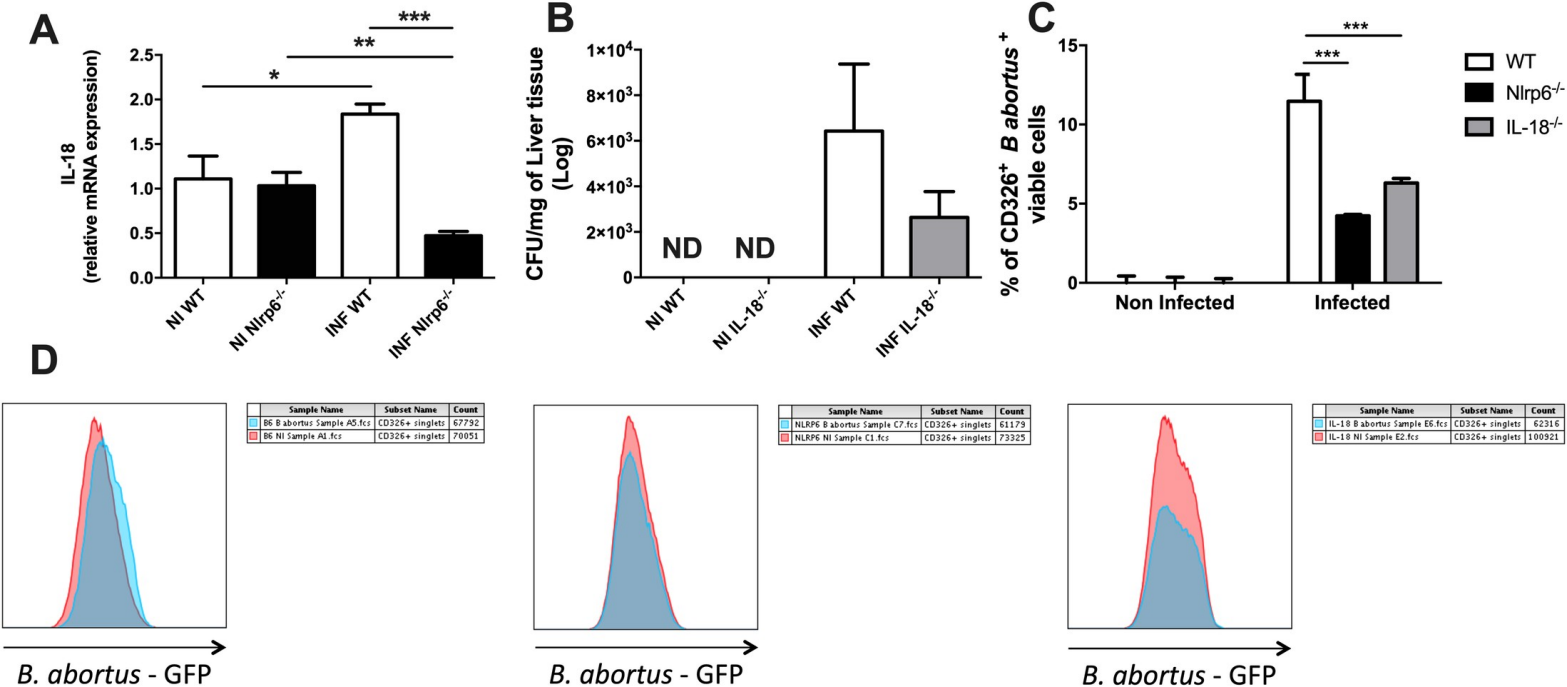
The cytokine IL-18 participates in the *B*. *abortus* oral infection dependent on activation of NLRP6 inflammasome. **(a)** Nlrp6^-/-^ mice have lower expression of mRNA for IL-18 after oral infection by *Brucella abortus*. **(b)** Reduction of the bacterial load in the liver of deficient animals for IL-18. **(c and d)** WT mice had a higher percentage of viable intestinal epithelial cells infected. Times of 0h (non-infected animals) and 3 days were compared. Results were expressed as mean ± standard error of mean (n = 5–7). For the data of the epithelial cell infection experiment mean ± standard error of the mean (n = 1). *p < 0.05; **p < 0.01; ***p < 0.001.

We proceeded to evaluate the role of NLRP6 in deficient mice orally infected with *B*. *abortus*. After 3 days post-infection, a higher number of bacteria were recovered in the liver of WT mice when compared to Nlrp6^*-/-*^ mice (**Fig 2A**). *B*. *abortus* CFU amounts were also found reduced in the liver from Nlrp6^*-/-*^ mice at late time point (7 days after infection) compared to WT mice (**S2 Fig**). However, this difference was not observed when the bacteria were administered intraperitoneally (**Fig 2B)**. Moreover, a significant increase in intestinal permeability of WT infected mice was observed, in contrast with Nlrp6^*-/-*^ mice, showing no significant differences when compared to the uninfected group (**Fig 2C**). Since NLRP6 seems to be involved in the process of increasing intestinal epithelial permeability, we sought to assess which factors could be regulated in this process, evaluating the expression of *MUC-1* and *MUC-2* (mucin transcripts), important molecules for the production of mucus, the first defense layer of intestinal epithelium. We also evaluated the expression of *Amphiregulin* (AREG), important for the regeneration of intestinal epithelium and *IL-18*, the main molecule involved in the activation of antimicrobial peptides and in epithelial regeneration (**Figs 2D and 3A**). For *MUC-1* and *MUC-2* mRNA, we observed that the Nlrp6^*-/-*^ animals have a naturally lower expression of these transcripts, indicating that mucus layer production is compromised in the absence of NLRP6. For WT animals, oral infection by *Brucella abortus* induced a reduction in the expression of only *MUC-1* (**Fig 2E and 2F**). Regarding the expression of *Amphiregulin* and *IL-18*, the infection led to an increase in the transcripts of the two molecules in WT animals; however, in Nlrp6^*-/-*^ animals, only a reduction of *IL-18* was observed in the infected animals compared to the uninfected animals, and this reduction was significant when compared to infected WT animals. Together, the data indicate that an intact NLRP6 is required to maintain several mechanisms of mucosal homeostasis, although it does not impact the *B*. *abortus* gut infection.

### The gut inflammatory response differs between wild-type and NLRP6 deficient mice before and after *B*. *abortus* infection

The cytokine IL-18 is produced in the intestine via activation of NLRP6 by the intestinal microbiota. In this context, we also defined the role of IL-18 in *Brucella* infection in vivo using deficient animals for this cytokine (IL-18^-/-^), since the differential expression of this molecule in our experimental groups seems to be directly related to the maintenance of the epithelial barrier. Of note, a reduction of bacterial load in the livers of IL-18^-/-^ animals was observed although it was not statistically significant (**Fig 3B**). To evaluate the ability of *B*. *abortus* to infect intestinal epithelial cells in the presence or absence of NLRP6, the experiment was performed using WT, Nlrp6^*-/-*^ and IL-18^-/-^ animals. These mice were infected orally with the fluorescent bacteria (*B*. *abortus*-GFP) and after 3 days of infection the intestine was collected and the epithelial cells (CD 326+) were isolated by mechanical and enzymatic digestion. The cells were then evaluated for presence or absence of bacteria and viability by flow cytometry. As a result, WT animals presented greater percentage of infected viable epithelial cells than IL-18^-/-^ and Nlrp6^*-/-*^ groups, which presented similarly low rates of infected viable cells (**Fig 3C and 3D**).

**Fig 4:**
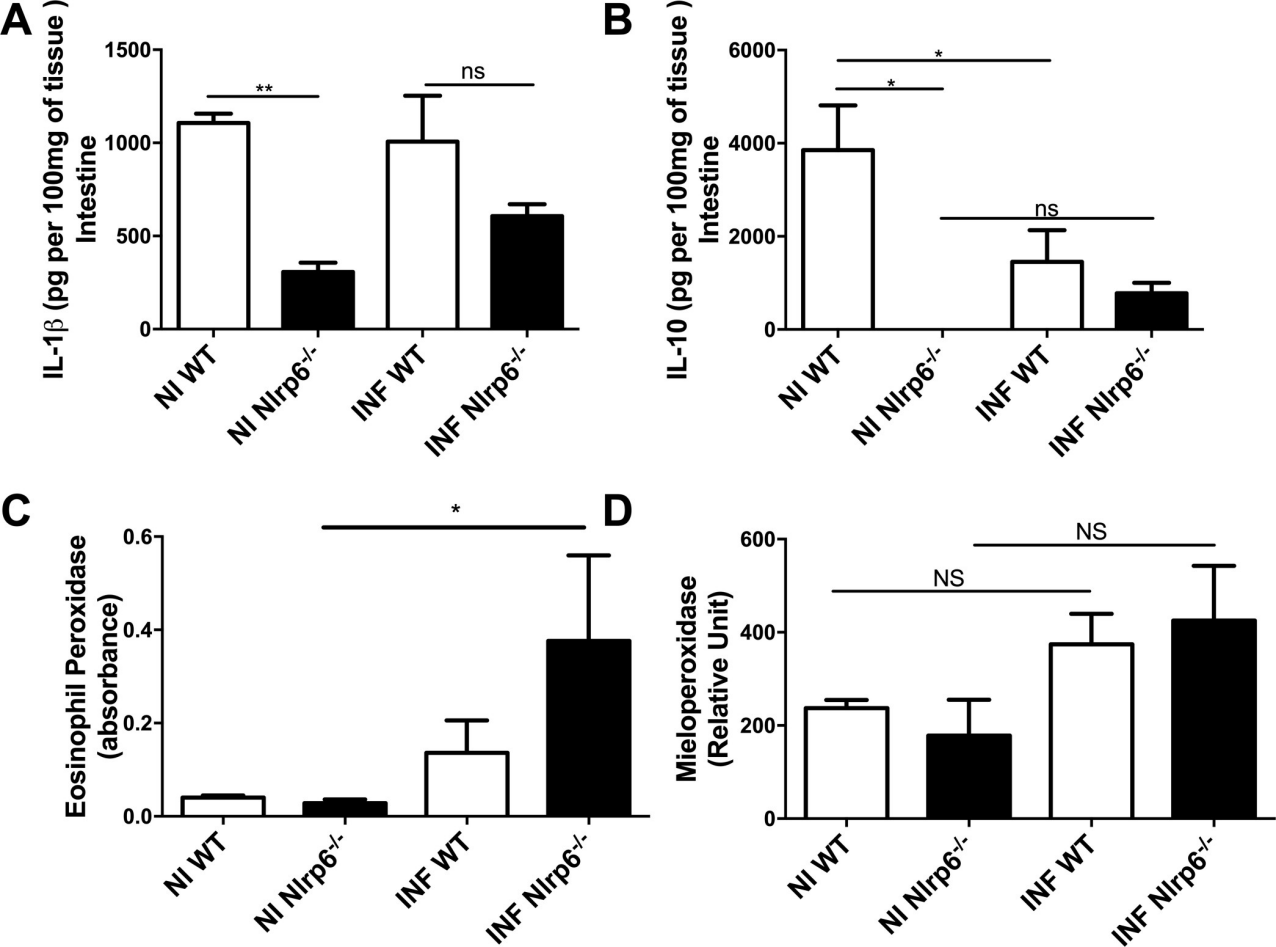
Evaluation of inflammatory parameters in WT *versus* Nlrp6^-/-^ mice after *B*. *abortus* infection. **(a)** Non-infected Nlrp6^-/-^ animals naturally have lower levels of cytokine IL-1β production. **(b)** Nlrp6^-/-^ animals not infected have naturally lower levels of cytokine IL-10 production. **(c)** Nlrp6^-/-^ animals have increased EPO after 3 days of infection. **(d)** There are no significant changes in MPO expression in the small intestine. Results expressed as mean ± standard error of mean (n = 5–7). *p < 0.05; **p < 0.01; ***p < 0.001.

Then, we analyzed whether other immune mechanisms involved in mucosal response were altered in Nlrp6^-/-^ mice. Lower levels of IL-1β and IL-10 were found in the gut of Nlrp6^*-/-*^ mice compared to WT mice, in both infected and no-infected groups (**Fig 4A and 4B**). Additionally, in order to investigate whether the inflammatory response could be one of the factors involved in the phenotype observed, we evaluated the infiltration of inflammatory cells in the small intestinal tissue: neutrophils and eosinophils by measurement of the MPO and EPO enzymatic assays, respectively. Intriguingly, we observed an increase in the expression of eosinophil peroxidase (EPO) in the tissue of Nlrp6^*-/-*^ mice when compared to the uninfected group (Fig 4C), and together with findings shown in Fig 1C and 1D, it suggests eosinophilic infiltration in the gut of infected mice (**Fig 4C**).

No significant difference in MPO was observed between WT and Nlrp6^-/-^ mice (**Fig 4D**).

### *B*. *abortus* oral infection altered the intestinal microbiota composition

**Fig 5:**
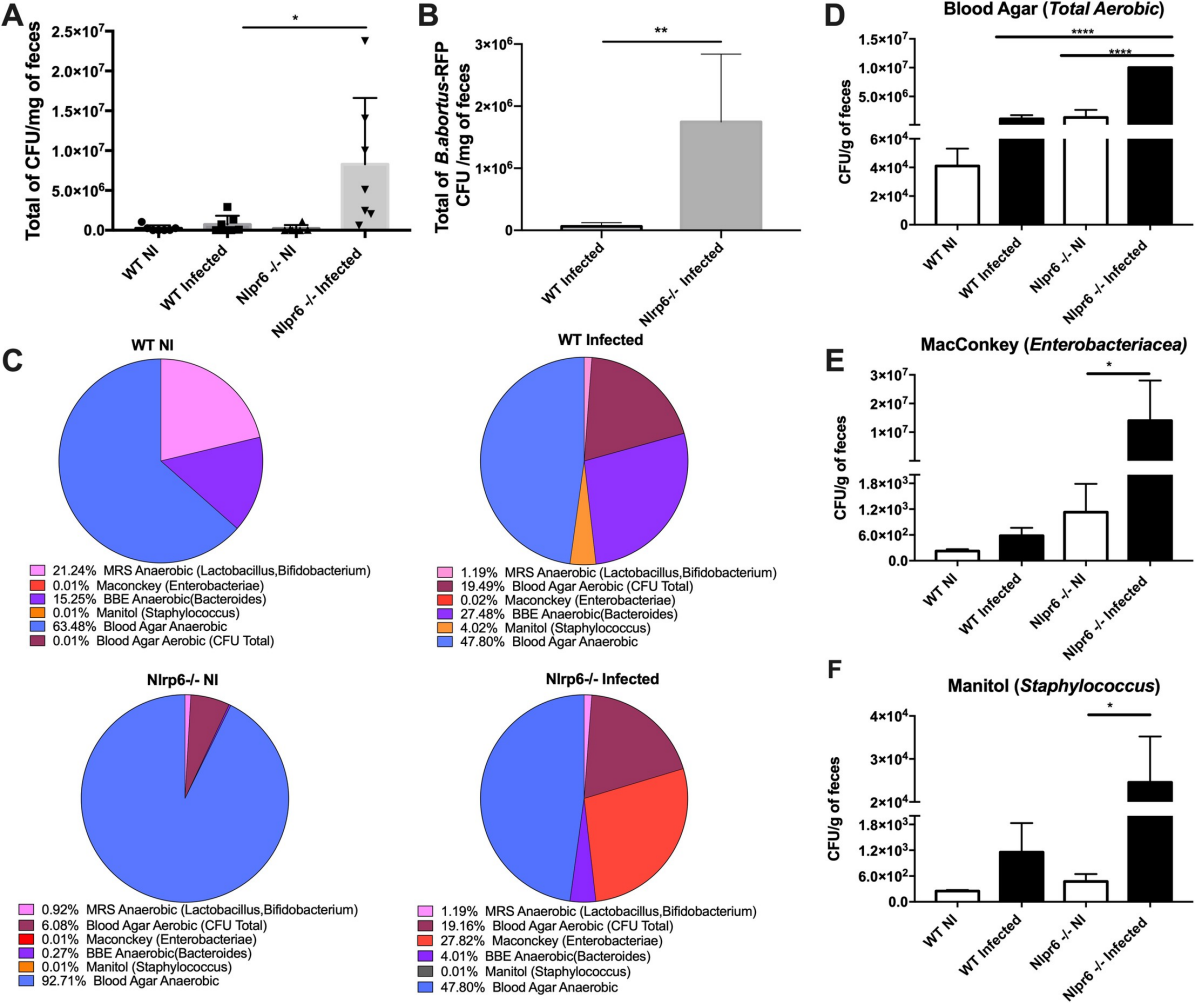
Quantitative and qualitative profile of the cultivable intestinal microbiota of WT and Nlrp6^-/-^ animals after oral infection by *Brucella abortus*. WT and Nlrp6^-/-^ animals were orally infected with 10^9^ CFU of *B*. *abortus* or fluorescent *B*. *abortus -RFP* and sacrificed after 0h (non-infected) and 3 days of infection. Feces were collected and cultivated in selective and/or differential culture media in the presence or not of oxygen at 37°C days. **(A)** Quantification of the total number of cultivable bacteria components of intestinal microbiota from WT and Nlrp6^-/-^ animals. **(B)** Quantification of fluorescent *B*. *abortus -RFP* in intestinal content from WT and Nlrp6^-/-^ animals. **(C)** Qualitative representation pie charts of the proportions between the groups of anaerobic and aerobic cultivable bacteria observed between the animal’s groups. Quantification of aerobic cultivable bacteria in: **(D)** blood agar, **(E)** Maconkey and **(F)** Manitol agar plates under aerobioses condition. Result expressed as CFU per mg of feces and as a percentage of representative groups of bacteria per experimental animal group. Results expressed as mean ± standard error of mean (n = 5–7). *p < 0.05; **p < 0.01; ***p < 0.001.

Since several mechanisms of gut barrier function were defective in Nlrp6^*-/-*^ mice but did not interfere on the *B*. *abortus* infection susceptibility, we proceeded to analyze whether the gut microbiota was involved in the *B*. *abortus* oral infection. WT and Nlrp6^*-/-*^ animals were infected by intragastric gavage, and after 0 and 3 days, representative collection of the entire intestinal content was performed for microbiota analysis through the culture-dependent method. Considering the total number of viable cultivated bacteria, it was observed that Nlrp6^*-/-*^ infected animals showed an increase in CFU when compared to infected WT mice **(Fig 5A)**. When comparing uninfected to infected animals, both WT and Nlrp6^*-/-*^ animals showed an increase in aerobic bacteria count after oral infection, suggesting that this increase could be due to the *Brucella* itself used in oral infection (**Fig 5B and 5C)**. Indeed, we observed increase CFU load of *B*. *abortus* in both WT and Nlrp6^*-/-*^ infected mice although larger amounts were found in the Nlrp6^*-/-*^ infected animals **(Fig 5B)**. However, evaluating different bacterial groups, through culture in selective agars, we found a greater diversity of intestinal microbiota components in uninfected WT animals when compared to uninfected Nlrp6^*-/-*^, although the latter group presents a greater CFU of anaerobic bacteria (**Fig 5C)**. In turn, when infected, the Nlrp6^*-/-*^ group presented an increase in the CFU of enterobacteria (MacConkey Agar), a characteristic increase observed predominantly in cases of dysbiosis **(Fig 5C and 5E).** When infected, WT animals presented the highest CFU of bacteria of the group *Staphylococcus* sp. (Mannitol Agar) (**Fig 5C and 5F)** and a reduction in anaerobic bacteria, such as *Lactobacillus* sp. and *Bifidobacterium* sp. (MRS Agar), important commensal bacteria components of a healthy intestinal microbiota (**Figs 5C and S3)**. These data suggest that oral infection by *Brucella abortus* leads to a significant alteration in the intestinal microbiota of infected animals and, interestingly, the alteration occurs in different bacterial groups when comparing the WT and Nlrp6^*-/-*^ animals.

### Gut microbiota plays a role in the intestinal epithelium barrier maintenance after *B*. *abortus* infection

**Fig 6:**
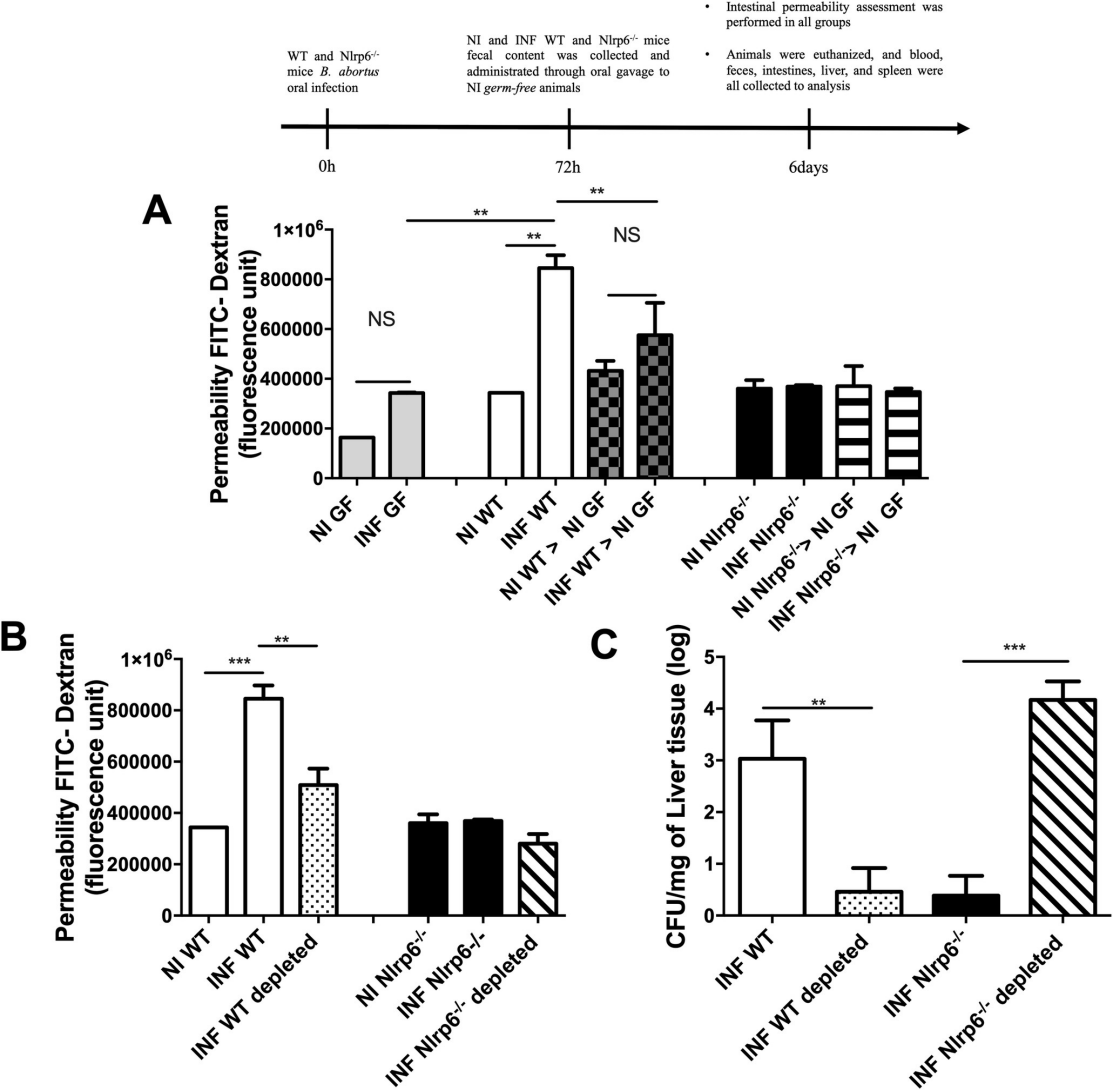
Mice microbiota is involved in the role of maintaining intestinal permeability and susceptibility to infection. **(a)** Germ-free (GF) recipients of intestinal microbiota from infected WT animals showed increased intestinal permeability. **(b)** Infected WT animals showed lower intestinal permeability in the absence of their microbiota (depletion). **(c)** Microbiota depletion reduces the bacterial load present in the liver tissue of WT animals’ concomitant to the increased concentration of *B*. *abortus* in animals deficient for NLRP6. Results expressed as mean ± standard error of mean (n = 5–7). *p < 0.05; **p < 0.01; ***p < 0.001. NI = Non infected.

As previously observed, *B*. *abortus* infection led to increased intestinal permeability of WT animals, which did not occur in Nlrp6^*-/-*^ mice. In order to elucidate the importance of intestinal microbiota for the observed phenotype, WT and Nlrp6^*-/-*^ animals were infected orally and after 3 days their feces were collected aseptically, processed and administered to germ-free (GF) mice by oral gavage. After 3 days of transplantation, intestinal permeability was again evaluated. As a result, it was observed that the GF animals which received the microbiota from the infected WT animals, also showed increased intestinal permeability. Similarly, GF animals that received the intestinal microbiota from infected or uninfected Nlrp6^*-/-*^ animals did not present changes in intestinal permeability. A statistical difference was also observed between the group of infected WT animals and the group of GF animals that received their feces. Finally, infected GF animals did not present significant change in intestinal permeability (**Fig 6A**). Knowing that microbiota is involved in the maintenance of gut permeability, we performed microbiota depletion of WT and Nlrp6^*-/-*^ animals through the use of a wide spectrum antibiotic cocktail for 21 consecutive days. After confirmation of depletion through sterility test, the animals were infected with *B*. *abortus* and, after 3 days of infection, the intestinal permeability test and bacterial count in liver tissues were performed. In WT microbiota-depleted mice, the *B*. *abortus* infection was no longer able to alter the intestinal permeability, unlike the phenotype previously observed. In addition, the microbiota abrogation in WT mice reduced the *B*. *abortus* CFU load in the liver compared to infected conventional WT mice. Nlrp6^*-/-*^ mice showed no alteration in the intestinal permeability after *B*. *abortus* infection, but surprisingly, when the microbiota was depleted in these mice, there was a significant increase in the *B*. *abortus* CFU load in the liver after oral infection with *B*. *abortus* (**Fig 6B and 6C**), indicating that the resistant phenotype observed in Nlrp6^*-/-*^ infected mice was dependent on the gut microbiota composition.

### NLRP6 regulates Immunoglobulin A (IgA) secretion after *B*. *abortus* infection

**Fig 7:**
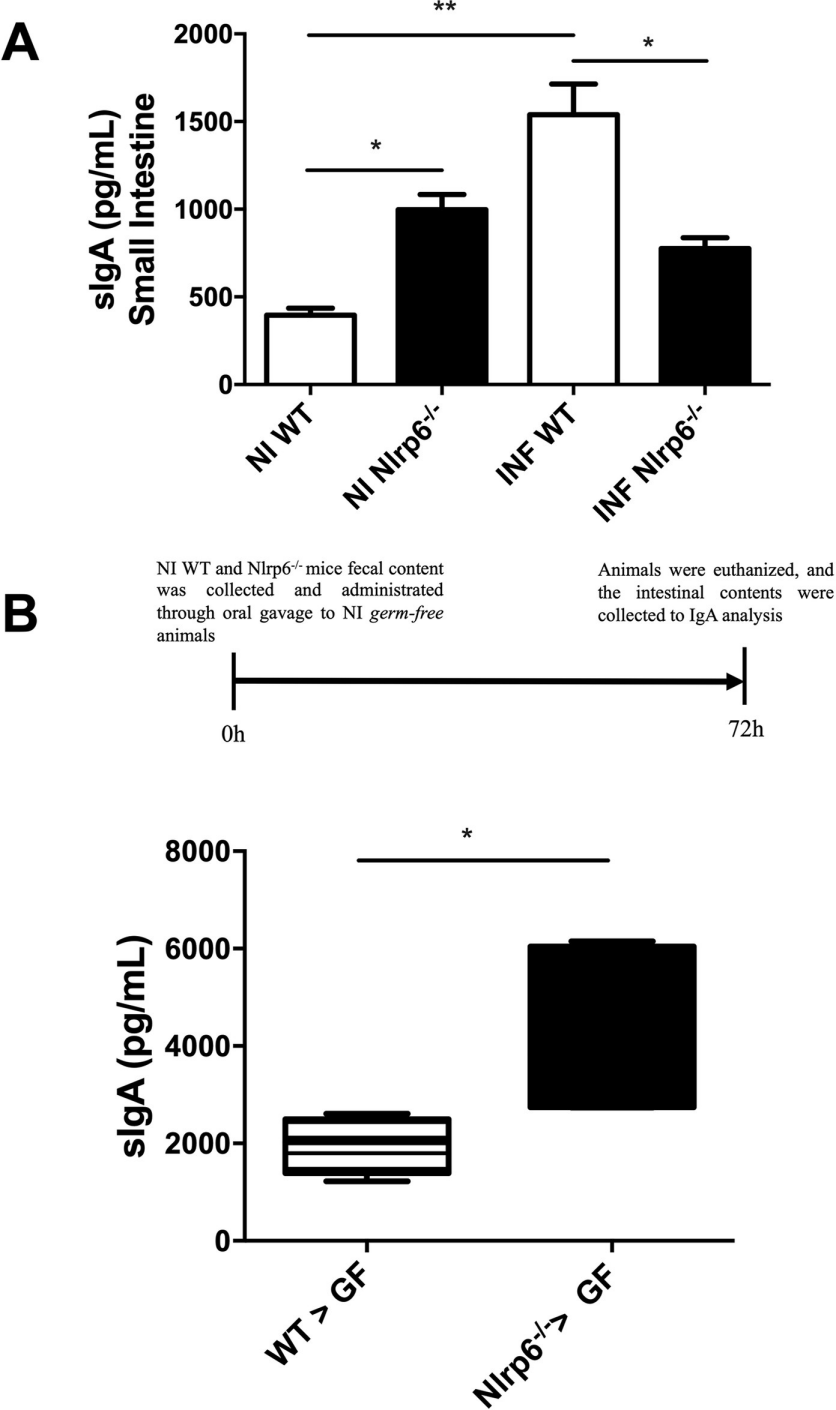
Immunoglobulin A secreted (SIgA) levels in WT versus Nlrp6^-/-^ mice. **(a)** Nlrp6^-/-^ animals have no changes in IgA secretion levels after oral *Brucella abortus* infection. **(b)** Germ-free animals that received the intestinal microbiota from Nlrp6^-/-^ animals had higher levels of IgA secretion. Results expressed as mean ± standard error of mean (n = 5–7). *p < 0.05; **p < 0.01; ***p < 0.001.

Secreted immunoglobulin A (sIgA) classically acts as one of the first lines of defense in the protection of the intestinal epithelium against pathogenic and enteric toxic microorganisms and is one of the component mechanisms of the intestinal epithelial barrier [9]. For this reason, the evaluation of IgA secretion is important in the face of infection by the *B*. *abortus*. After oral infection, increased secretion of IgA was observed in Nlrp6^*-/-*^ animals compared to uninfected WT. In contrast, the Nlrp6^*-/-*^ infected animals did not show an increase in the secretion of IgA compared to uninfected Nlrp6^*-/-*^ mice. Interestingly, in the absence of infection, the Nlrp6^*-/-*^ animals demonstrated to have higher levels of sIgA in relation to WT animals (**Fig 7A**). The measurement of sIgA of the intestinal fluid of the animals submitted to the microbiota fecal transplantation experiment was also performed. As a result, it was observed that the GF animals that received the microbiota from WT animals secreted a lower amount of sIgA than the GF animals, which received the microbiota from Nlrp6^*-/-*^ animals (**Fig 7B**). Thus, it is possible to infer that NLRP6 has a regulatory role in sIgA secretion, mainly via regulation of intestinal microbiota.

## Discussion

Brucellosis can be transmitted by several routes, although the natural infection in humans and in other animals seems to occur by the oral route. Despite being a site of supposed importance during the invasion of the bacterium, there are few studies that address the gastrointestinal tract in the context of brucellosis.[13,20–25]. The major findings of this study can be summarized as follows: i*) B*. *abortus* administered orally induced intestinal barrier leak and microbiota dysbiosis, which correlates temporarily with the CFU levels in the liver; ii) the Nlrp6^*-/-*^ mice without the inflammasome NLRP6 were more resistant to the *B*. *abortus* when orally infected and; iii) we also provide novel findings showing that the gut permeability phenotype induced by *B*. *abortus* after early stages of oral infection is partially associated with gut microbiota modulation.

The intestinal mucosal epithelium acts as a sentinel for recognition and initiation of immune responses against bacteria [26]. One of the invasion pathways of the *B*. *abortus* is through the intestinal epithelial cells [27], however this mechanism has yet to be fully explained. In the present study, it was found that the breaking of epithelial barrier 3 days after the infection—observed by the increase in intestinal permeability in WT mice–which was associated with increase tissue damage and inflammation in the gut. The CFU of bacteria in the liver was found increased in early time points, which could be explained by the fact that liver is a firewall that capture gut bacteria through the blood during intestinal pathology [1]. Taken together, our result suggested that the breaking of epithelial cell barrier after infection could be a potential mechanism that would facilitate the entry of the pathogen in this model of infection. Although Paixao *et al* [25] have also established the oral infection with other *Brucella* species (*Brucella melitensis*), they did not find any physiological alteration in the gut, as opposed to those found in these histopathology experiments. This is due to the fact that different bacterial species are being used in this study, forcing various pathogens to use distinct mechanisms and molecules in the intestinal invasion process [28]. One of these mechanisms was explored in this study, the inflammasome NLRP6, albeit other inflammasome molecules, such as AIM2 and NLRP3, have already been shown to be important in controlling *B*. *abortus* infection systemically [29]. Meanwhile, the NLRP12 inflammasome, in turn, has been suggested as important in the negative regulation of the initial inflammatory response against *B*. *abortus*, since Nlrp12^-/-^ mice were more resistant, at early stages, but again, by the intraperitoneal route [30]. It is worth pointing out that studies in humans and mice have found high expression of NLRP6 in intestinal epithelial cells [16,17,31]. It was possible to observe that the lack of NLRP6 was important in the model of *Brucella* infection, but only when the infection was administered via oral route, not through the intraperitoneal administration. The findings obtained here, showing that epithelium cells harvested from wild-type mice were more susceptible compared to Nlrp6^*-/-*^, suggest the ability of *Brucella* to infect gut epithelium cells, but also confirm the determinant role of NLRP6 in this cell type that is located in the intriguing host-microbiota interface. IL-18 is regulated by NLRP6 inflammasome activation, and their importance to the epithelium regeneration has been already described [19]. Here, it was also found that IL-18 is involved in *B*. *abortus* oral infection, observed by the increase levels of mRNA in the gut of wild-type infected mice but not on Nlrp6^-/-^ infected animals.

Elinav et al. (2011) [16] already demonstrated that the NLRP6 inflammasome present in the colon epithelium plays an important role in regulating the microbiota. They observed that the deficiency of NLRP6 results in drastic consequences in the composition of the microbial community, leading to dysbiosis through the expansion of potentially pathogenic bacterial groups. In addition, as seen in other studies, NLRP6 deficiency led to a reduction in the activation of caspase-1 and IL-18, which are fundamental for intestinal homeostasis [19,32]. Corroborating previous studies, in uninfected animals, these experiments found less diversity in the Nlrp6^*-/-*^ gut microbiota when compared to wild-type mice. Furthermore, total gut microbiota CFU numbers were significantly higher in the Nlpr6-/- infected mice compared to the wild-type infected mice. Also, we found higher CFU loads of *B*. *abortus* suggesting that it persisted in the intestinal lumen and may be related with the reduction of CFU in the liver associate with the resistance phenotype observed in the Nlpr6^-/-^ mice. However, the intestinal higher CFU load found in Nlpr6-/- infected mice is not exclusive to *B*. *abortus*. Another explanation might be related to the growth of other bacteria groups belonging to the gut microbiota, for example, the enterobacteria group, closely associated with dysbiosis. Indeed, our results demonstrated, for the first time, that even in wild-type and Nlrp6^-/-^ mice the *B*. *abortus* oral infection leads to dysbiosis, characterized here by reduction of anaerobic bacterial cultivable groups, such as Bifidobacterium, Lactobacillus and Bacteroides, all well recognized as beneficial to gut homeostasis [10,12,33]. However, we do recognize that using large inoculum numbers of *Brucella abortus* to optimize the experimental model of oral infection, might be directly involved in the modulation of gut microbiota. Thus, we found it to be an important limitation in this work.

Several studies have proposed that the gut microbiota dysregulation is involved in the rupture of the gut epithelium barrier [10,12,34]. Here, it was found that the breaking of the epithelial barrier *in vivo* might be associated with the presence of the NLRP6 receptor, since in the deficient mice, no change in intestinal permeability was observed after infection. In addition, the changes in the gut permeability, especially in wild-type mice, were mediated by the gut microbiota alteration and could be one of the mechanisms that facilitate the entry of *B*. *abortus* in this infection model. Supporting this hypothesis, antibiotic-induced microbiota depletion in WT mice led to changes in intestinal permeability and reduced the systemic bacterial load. In turn, the gut permeability was not altered in Nlrp6^*-/-*^ infected or uninfected deficient mice. It should be noted that although the gut microbiota depletion on Nlrp6^*-/-*^ mice did not alter the gut permeability, higher *B*. *abortus* CFU numbers in the liver of those mice were observed. Thus, suggesting that the microbiota is exceptionally important for the protection of those mice against the *B*. *abortus*, and that the rupture of the gut epithelial does not seem to be the primary factor for *Brucella* invasion. It would also be an indication that NLRP6 is important for the control of *B*. *abortus* survival. Besides, as suggested by Elinav (2011), the unnatural dysbiotic (pathobiont) gut microbiota from Nlrp6^*-/-*^ mice may also be important to the direct competition mechanism against *B*. *abortus*, consequently interfering in their host invasion. In fact, many bacteria from the microbiota directly inhibit pathogens, due to competition for nutrients, for example. This induces the production of inhibitory substances by the symbionts, such as bacteriocins, which act directly on pathogens, as demonstrated for example in *C*. *difficile* and *Listeria* sp. In our study, however, this mechanism is not explored [35–37].

Additionality, the higher levels of sIgA in the intestinal contents from uninfected Nlrp6^*-/-*^ compared to uninfected wild-type mice reinforce that the gut microbiota composition may be involved in the regulation of sIgA. SIgA classically performs the process of immune exclusion by promoting the clearance of antigens in the intestinal lumen, facilitating its removal by muco-ciliary activity [8,9]. Also, worth noting is that the *B*. *abortus* infection induced the sIgA levels in the gut of WT mice only, suggesting that the NLRP6 molecule itself may have a regulatory role in the production and/or secretion of sIgA.

Other characteristics observed from the dysbiotic microbiota that colonized the Nlrp6^*-/-*^ mice is the ability to transfer their phenotype to wild-type mice [16,38]. It was also observed in the present study that when transferring the microbiota of the mice from both groups, which had previous contact with *B*. *abortus*, to germ-free mice, they established a gut permeability phenotype similar to that of the respective donors. Besides, the maintenance of this phenotype does not seem to be directly related to the gut bacterial load of *B*. *abortus*, since higher CFU counts were found in feces of Nlrp6^*-/-*^ infected mice. Of note, germ free (GF) mice was used to elucidate the importance of gut microbiota on the gut permeability, and infected GF did not change gut permeability whereas GF that received feces from WT infected mice increased the permeability, suggesting that, not only the composition but also the gut microbiota presence itself have a role on breakdown of the intestinal epithelial barrier. We do recognize that non-use of littermates of WT and Nlrp6^-/-^ mice is a limitation of the study. To further investigate the role of *B*. *abortus* infection in differences in gut microbiota of Nlrp6^-/-^ mice, littermates should be used in future studies.

When exploring other mechanisms related to the epithelium barrier integrity, we found that there was an increased expression of amphiregulin (AREG) only in the intestine of WT, but not in Nlrp6^-/-^, after infection with *B*. *abortus*. Furthermore, amphiregulin is known to be important for regeneration of the epithelium after injury [38]. This finding suggested that NLRP6 may influence the epithelium integrity through another mechanism. Moreover, we found that the *B*. *abortus* oral infection did not alter the transcription of mucin (MUC 1 and MUC 2) in both wild-type and Nlrp6^*-/-*^ mice. However, steady state Nlrp6^*-/-*^ mice already presented reduction on the expression of these molecules, thus, suggesting that the dysbiotic microbiota in Nlrp6^*-/-*^may be involved in the reduction of mucus production on those mice, as previously described by another group [17].

During infections, the inflammatory response may be induced to control the infectious foci. There are reports in clinical literature showing gastrointestinal inflammation caused by *Brucella* in humans, resulting in, for example, colitis and ileitis [39–42], suggesting a gastrointestinal inflammatory process participating in the infection. In the presented study, significant increase levels of inflammatory mediators, such as cytokines, were not found, suggesting that the *B*. *abortus* oral infection model is quite “silent”. However, it was shown for the first time that the granulocytes inflammatory cells, especially eosinophils, were elevated in the gut of infected wild-type mice. This finding corroborates rare reports in medical literature that present eosinophilia as a clinical manifestation in patients with brucellosis [43,44]. Albeit high levels of eosinophils were found on the Nlrp6^-/-^ mice, both in the gut and blood, this increase may be associated with a greater intestinal defense against the invasion of *B*. *abortus*. Since eosinophils can act as effector cells, releasing toxic granules and extracellular DNA traps directly into the pathogen, and also on tissue remodeling/ repair of epithelium cells, their presence in Nlrp6^-/-^ infected mice may be contributing to the phenotype of resistance observed in these animals [45,46]. From a different perspective, Anand et al. (2012) [47] observed that NLRP6-deficient animals were more resistant to infection by *Listeria monocytogenes*, *Salmonella* Typhimurium and *Escherichia coli*, showing an increase in the number of monocytes and circulating neutrophils, in addition to an increase in cell production of cytokines and chemokines dependent on NF-κB and MAPK pathways. Previous studies also corroborated our findings, suggesting the presence of NLRP6 molecule as a negative regulator of *B*. *abortus* gut infection. Our findings highlight the potential of NLRP6 protein as a therapeutic drug target in *Brucella abortus* infection, in addition to the role of host microbiota in modulating the epithelium barrier function. The molecular mechanisms underlying the role of NLRP6, associated or not with the gut microbiota dysbiosis, induced by *B*. *abortus* oral infection, need to be further investigated.

## Conclusion

This study has shown that oral infection by *Brucella abortus* not only alters intestinal homeostasis, favoring its invasion in the intestine epithelium and the establishment of systemic infections, but is also dependent on gut microbiota composition. In turn, by using two mouse colony strains, with different gut microbiota composition, we conclude that the resistance phenotype to *B*. *abortus* is conferred by the gut microbiota, and that the resistance is not mediated by NLRP6 inflammasome molecule.

## Materials and methods

### Ethics statement

The experimental procedures performed were previously approved by the Ethics Committee on the Use of Animals of the Federal University of Minas Gerais—CEUA/UFMG under protocol number 273/2017.

### Experimental animals

Wild-type C57BL/6, Nlrp6^*-/-*^, IL-18^*-/-*^, and Swiss from eight- to twelve-week-old mice were used in this study. Germ-free mice were also used, kept in flexible Trexler isolators (Madison, EUA), transferred to micro-isolators to experimental procedures and handled according to pre-established sterile techniques. All mice were sustained in a free-pathogens animal facility (Laboratory of Immunology of Infectious Diseases–Institute of Biological Sciences/UFMG), in a controlled climate with free access to water and food.

### RNA isolation and quantitative real-time PCR (qPCR)

The intestinal tissue was harvested following euthanasia. and samples were immediately stored in N_2_ for further use. Total RNA was isolated by Trizol reagent (Invitrogen), accordingly to manufacturer instructions. After DNase I treatment, 1 μg of each total RNA was reverse transcribed using 500 ng of oligo dT primer and the resulting cDNA was used as template for qPCR. The reaction was performed in a final volume of 10 μl containing SYBER Green PCR Master Mix (Thermo Fisher Scientific, USA), cDNA, and 5 μM of specific primers for the gene of interest (murine GAPDH, MUC1, MUC2, IL-18, and Amphiregulin), in an ABI/PRISM 7000 Sequence Detection System (Applied Biosystems, Foster City, CA). Data were analyzed with the Threshold Cycle (ΔΔ^Ct^) method and they were presented as relative expression units after normalization to GAPDH housekeeping gene. All reactions were performed in triplicate.

### Bacterial strain and *in vivo* infection

The bacteria *B*. *abortus* strain 2308 and the fluorescent *B*. *abortus*- RFP obtained from our collection were grown in Brucella Broth medium (BB, BD Biosciences, EUA) under appropriated conditions[48], washed in PBS and resuspended in PBS. Mice were orally infected by intragastric gavage technique with 100 μL of bacterial suspension containing 1x10^9^ colony forming units (CFU) of *B*. *abortus* or PBS (control group). To systemic infections, mice were inoculated intraperitoneally (i.p.) with 100 μL containing 1x10^6^ CFU of *B*. *abortus* or PBS. After three days of infection, spleen and liver of all mice were homogenized with saline solution (1 mL saline per 100 mg tissue), serially diluted 10-fold and plated on BB agar plates, to enumerate viable bacteria. CFUs were counted after 3 days of incubation at 37°C and results are shown as mean ± SEM of CFU/milligram of tissue.

### In vivo intestinal permeability assay

*In vivo* intestinal permeability to assess barrier function was measured as previously described. Briefly, mice were starved for 4 hours prior to experiments. The fluorescent-labelled FITC-Dextran was orally gavage at a dosage of 600 mg/kg. Blood samples were obtained after 4 hours of administration, and the fluorescence intensity in the serum was measured at an excitation wavelength of 490 nm and an emission wavelength of 530 nm using a spectrophotometer [49]. The intestinal permeability was expressed as a mean ± SEM of fluorescence units per group.

### Histopathologic assay

For histopathological examination, small intestine and colon were removed from each mouse, and tissues were emptied of its luminal contents by squeezing, avoiding damage to the mucosa. Each tissue was coiled into a small roll and fixed in Bouin’s solution, with 2% of glacial acetic acid. The samples were fixed by immersion in 10% formalin solution in PBS, cut into small sections of 4-μm-thick, stained with hematoxylin-and-eosin (H&E) [49,50] and examined under light microscopy by a pathologist blinded to the experiment. Measurement of villus heights was performed using the ImageJ software. The histopathology score (inflammatory infiltrate) was measured as follow (maximum score 3–0 no inflammation, 1 –slight infiltration of inflammatory cells, 2- moderate infiltration of inflammatory cells, 3- large infiltration of inflammatory cells also in submucosa). Ten intact and well-oriented microscopic-sections were measured from each animal of each mouse group (n = 5).

### Intestinal microbiota analysis

Luminal contents and feces from the small intestine, cecum, and colon were collected aseptically, weighted and serially diluted in PBS before plating on agar plates. The enriched, selective or differential media were used as follows: Mannitol Salt Agar to isolate *Staphylococcus*; MacConkey Agar (MAC) to select Enterobacterium; Brucella Broth (BB) to cultivate *Brucella* spp. and other microorganisms; Brain Heart Infusion Agar (BHI) added with Sodium Azide to isolate *Enterococcus*; Blood Agar provides conditions to the growth of several microorganisms, including *Streptococcus* spp.; MRS Agar to cultivate *Lactobacillus* and *Bifidobacterium*; Bacteroides Bile Esculin (BBE) to cultivate Bacteroides. Plates were incubated under aerobic and anaerobic conditions at 37°C. After 2–4 days, CFU were counted and the results were expressed as a mean ± SEM of CFU/mg of feces. For *Brucella abortus*-RFP, CFU determination, the colonies expressing red coloration were counted and the result also expressed as CFU/mg of feces.

### Fecal microbiota transplantation: Conventionalization of germ-free mice

Conventional WT and Nlrp6^-/-^ were previously infected orally with *B*. *abortus* and 3 days later, the fecal content was aseptically collected and diluted with PBS [50]. One hundred microliters of the suspension were administrated through oral gavage to *germ-free* animals. Three days after procedure, animals were euthanized, and blood, feces, intestines, liver, and spleen were all collected to analysis as described previously.

### Gut microbiota depletion

Gut microbiota was depleted in WT and Nlrp6^-/-^ mice with a broad-spectrum cocktail of antibiotics as previously described [51]. Initially, amphotericin B (0.1 mg/ml, every 12 hours) was administrated by oral gavage for 3 days, and subsequently, drinking water was supplemented with a cocktail composed of ampicillin (2 g/L), neomycin (2 g/L), metronidazole (1 g/L), and vancomycin (0.5 g/L) for 21 days. Additionally, all mice received ciprofloxacin i.p. (0.2 g/L). Feces were then collected and bacterial depletion were determinate on Thioglycolate medium, as described before [51]. Only mice with <1 CFU/mg feces were considered as depleted and used for further experiments.

### Myeloperoxidase and eosinophil peroxidase enzymatic assay

To measure myeloperoxidase (MPO) activity, snap-frozen colon and small intestine samples were made up to 100 mg/mL with PBS containing protease inhibitors, homogenized, centrifuged at 3000g for x 10 min at 4°C, and lysed. Samples were centrifuged again and resuspended in a buffer containing hexadecyltrimethylammonium bromide (HTAB, 0.5% w/v) and re-homogenized, before being transferred to microcentrifuge tubes of 1,5mL and submitted to 3 freeze-thaw cycles by immersing tubes in liquid nitrogen, as described before [52]. Samples were centrifuged once again, and supernatant was collected and kept on ice until further analysis. MPO activity was calculated by measurement of optic density [3] alteration at 450 nm using tetramethylbenzidine (TMB) using microplate reader (Multiskan FC Thermo Scientific, EUA). Eosinophil peroxidase (EPO) was also measured to estimate intestinal eosinophilia. Colon and small intestine tissues were made up to 100 mg/mL with PBS 5X (pH 7.2) and then homogenized using ultra-turrax, centrifuged and the supernatant discarded. The sediments were submitted to hypotonic lysis and then centrifuged one more time. Supernatants were discarded and the pellet was resuspended in 1,0mL of PBS (pH 7.4), containing HTAB. 1mL of this solution was submitted to 3 phases of freezing using liquid nitrogen. Samples were centrifuged again, and EPO activity was measured from supernatants as described previously at a wavelength of 492 nm [53].

### Cytokines measurement

Fragments of small intestine and colon (100 mg each) from WT and NLRP6^-/-^ mice were weighted and homogenized with 1 mL lysis buffer (0.1mM PMSF, 0.1mM benzethonium chloride, 10 mM EDTA and 20 KI of aprotinin A and 0.05% Tween-20 in PBS) using ultra-turrax (T10 basic ULTRA-TURRAX, IKA, Germany), After centrifugation at 2000g for 10 min at 4°C, supernatants were immediately collected and stores at -80°C until further analysis. Measurement of cytokines IL-1β and IL-10 was performed using the ELISA DuoSet (R&D Systems, EUA), according to manufacturer’s protocol.

### Intestinal epithelial cells isolation and culture

Wild type, Nlrp6^-/-^ and IL-18^-/-^ mice were infected by intragastric gavage with *B abortus* GFP. Three days after infection, mice were euthanized, small intestines collected. Fat, blood vessels and mesenteric tissue were trimmed. Luminal contents were washed with DPBS cold buffer. The intestine was cut lengthwise and incubated in ice for 20 min in dissociation Reagent #1 [47 mL DPBS, 3 mL EDTA 0.5 M (30mM) (Sigma) 75 μL DTT 1 M (1.5 mM) (Sigma)]. The tissue was then incubated in 10 mL of Dissociation Reagent #2 [47 mL DPBS, 3 mL 0.5 M (30 mM) EDTA (Sigma)] at 37°C for 10 minutes. Then the tube was agitated for 30 seconds to release the epithelium from the basal membrane. The solution was centrifuged at 1000 x g for 5 min at 4°C and cells collected at DPBS with 10% FBS After centrifugation the cells were resuspended in DPBS containing Liberase (0.13 mg/ml) (Liberase Lt Research Grade, Roche, Switzerland) and incubated for 10 min in a water bath at 37°C. Then, 10% FBS and 20 U/mL of DNAse I (GE Healthcare, USA) were added to the solution and filtered through a70 μm cell strainer (BD Bioscience). Cells were washed with DPBS and resuspended in RPMI prior to use.

### Flow cytometry analysis

Flow cytometry was performed using Attune Acoustic Focusing (Life Technologies, USA) and analyzed using FlowJo software (Tree Star, Ashland, USA). Non-specific antibody binding was blocked with Fc Block (1:30 dilution in PBS supplemented with 0.5% BSA). PE-Cy7-conjugated CD326/Ep-CAM was purchased from BioLegend (clone G8.8; 1:100 dilution). The concentration of cell suspensions was adjusted to 5 x 10^5^ cells per 100 ul.

### Statistical analysis

Data analysis was performed using GraphPad Prism software v5.03 (GraphPad Software Inc, San Diego, California, EUA). All the results were presented as a mean ± SEM, using one-way ANOVA + Student-Newman-Keuls post-test. Results were designated significant when the P-value (P) < 0.05.

## Supporting information

S1 Fig Dose response effects of Brucella abortus CFU oral infection. WT mice were orally infected with 3 different doses (10^10^, 10^9^, 10^8^) of *B*. *abortus* CFU and sacrificed after 72hours of infection. (A) Presence of viable bacterial load in the liver were quantified by culture-dependent plated in medium Brucella Broth medium, incubate at 37°C for 48hours. (B) Myeloperoxidase assay (MPO) were measured in the small intestine tissue as an indirect enzymatic and quantitative assay to evaluated neutrophils infiltration. (A) Results are shown as mean ± SEM of CFU/mg of liver tissue.
(DOCX)

S2 Fig Evaluation of *B*. *abortus* CFU in the liver of WT versus Nlpr6-/- mice at two different time points. WT and Nlrp6^-/-^ animals were orally infected with 10^9^ CFU of *B*. *abortus* and sacrificed after 0h (non-infected -WT NI)) 3 days, and 7 days of infection. Presence of viable bacterial load in the liver were quantified by culture-dependent plated in medium Brucella Broth medium, incubate at 37°C for 48hours. Results are shown as mean ± SEM of CFU/mg of liver tissue.
(DOCX)

S3 Fig Bacterial CFU quantification by selective culture-dependent medium during *Brucella abortus* orally infection. WT or Nlrp6^*-/-*^ mice received a single oral dose of *B*. *abortus* and 3 days after, microbiota evaluation was assessed in feces. Samples were plated onto Blood agar (Aerobic) (A), Mannitol agar (B), MacConkey agar (C), Brain-Heart infusion agar (D), BBE agar, MRS agar (F) and Blood agar (Anaerobic) (G). Samples were either incubated in aerobic (A-D) or anaerobic (E-G) atmosphere. Statistical analysis was performed by One-way ANOVA followed by Newman-Keuls’ post test. **** indicates p < 0.001.
(DOCX)
