# Supplementary material for: NLRP6-associated host microbiota composition impacts in the intestinal barrier to systemic dissemination of Brucella abortus
Source: PLoS Negl Trop Dis. 2021 Feb 22;15(2):e0009171. doi: 10.1371/journal.pntd.0009171 (PMC7932538; doi:10.1371/journal.pntd.0009171)

**S1 Figure: Dose response effects of Brucella abortus CFU oral infection.** WT mice were orally infected with 3 different doses (10^10^ ,10^9^ ,10^8^) of *B. abortus* CFU and sacrificed after 72hours of infection. (A) Presence of viable bacterial load in the liver were quantified by culture-dependent plated in medium Brucella Broth medium, incubate at 37°C for 48hours. (B) Myeloperoxidase assay (MPO) were measured in the small intestine tissue as an indirect enzymatic and quantitative assay to evaluated neutrophils infiltration. (A) Results are shown as mean ± SEM of CFU/mg of liver tissue.


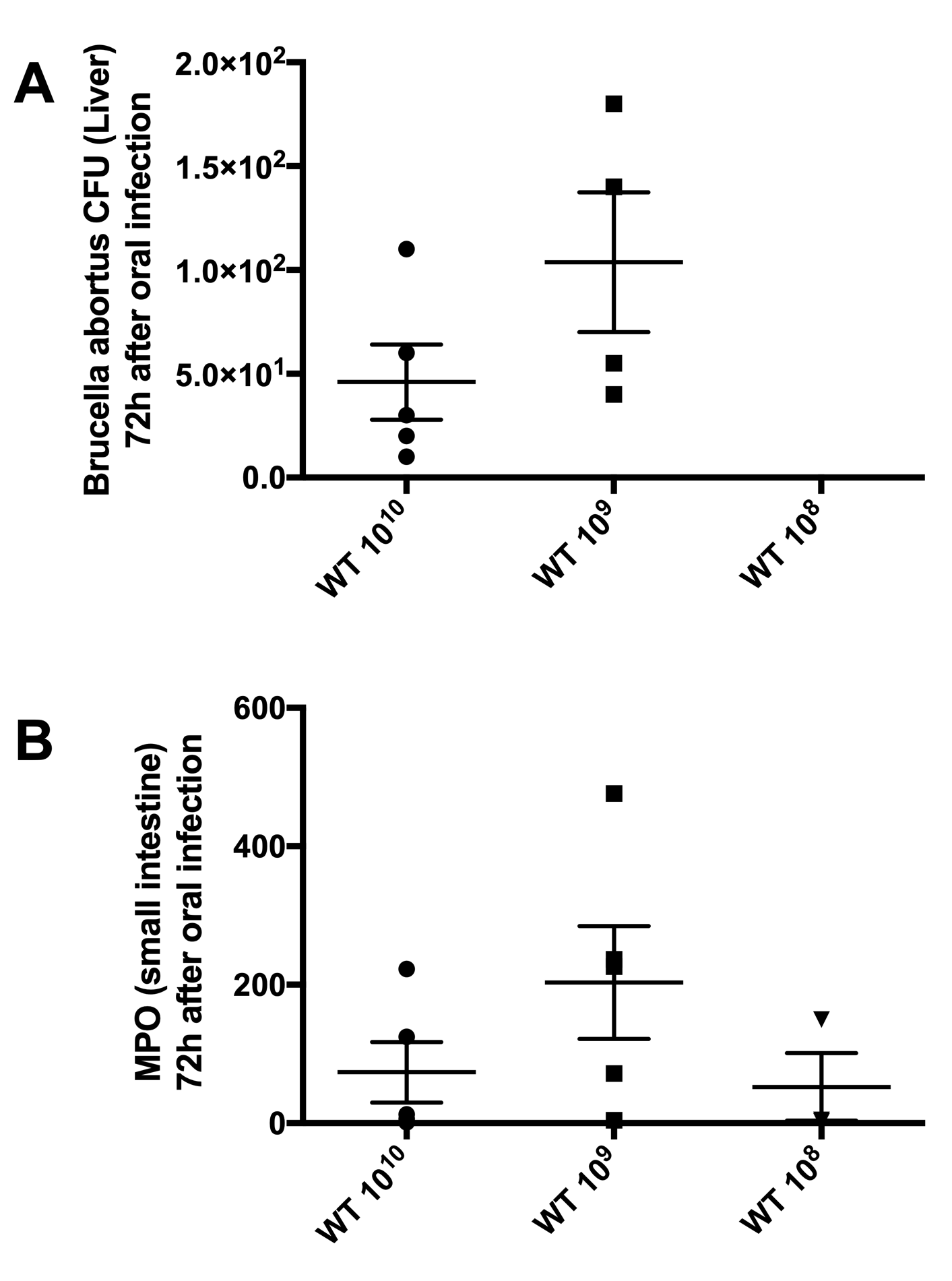

Supplement: S1 Fig — WT mice were orally infected with 3 different doses (1010, 109, 108) of B. abortus CFU and sacrificed after 72hours of infection. (A) Presence of viable bacterial load in the liver were quantified by culture-dependent plated in medium Brucella Broth medium, incubate at 37°C for 48hours. (B) Myeloperoxidase assay (MPO) were measured in the small intestine tissue as an indirect enzymatic and quantitative assay to evaluated neutrophils infiltration. (A) Results are shown as mean ± SEM of CFU/mg of liver tissue. (DOCX) [file pntd.0009171.s001.docx]
