# Supplementary material for: NLRP6-associated host microbiota composition impacts in the intestinal barrier to systemic dissemination of Brucella abortus
Source: PLoS Negl Trop Dis. 2021 Feb 22;15(2):e0009171. doi: 10.1371/journal.pntd.0009171 (PMC7932538; doi:10.1371/journal.pntd.0009171)

**S2 Figure.: Evaluation of *B. abortus* CFU in the liver of WT versus Nlpr6-/- mice at two different time points.** WT and Nlrp6-/- animals were orally infected with 10^9^ CFU of *B. abortus* and sacrificed after 0h (non-infected -WT NI)) 3 days, and 7 days of infection. Presence of viable bacterial load in the liver were quantified by culture-dependent plated in medium Brucella Broth medium, incubate at 37°C for 48hours. Results are shown as mean ± SEM of CFU/mg of liver tissue.


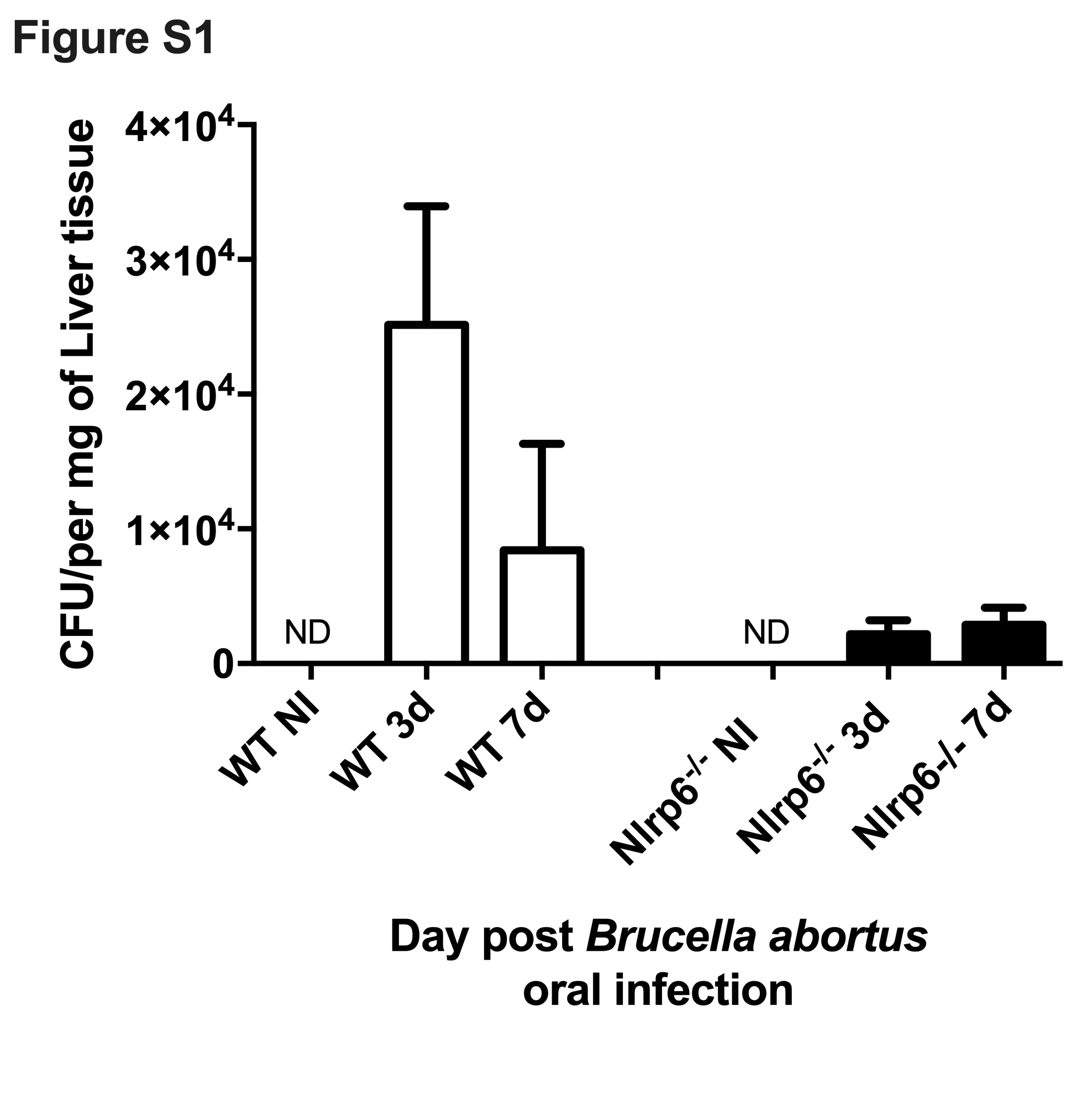

Supplement: S2 Fig — WT and Nlrp6-/- animals were orally infected with 109 CFU of B. abortus and sacrificed after 0h (non-infected -WT NI)) 3 days, and 7 days of infection. Presence of viable bacterial load in the liver were quantified by culture-dependent plated in medium Brucella Broth medium, incubate at 37°C for 48hours. Results are shown as mean ± SEM of CFU/mg of liver tissue. (DOCX) [file pntd.0009171.s002.docx]
