# Supplementary material for: NLRP6-associated host microbiota composition impacts in the intestinal barrier to systemic dissemination of Brucella abortus
Source: PLoS Negl Trop Dis. 2021 Feb 22;15(2):e0009171. doi: 10.1371/journal.pntd.0009171 (PMC7932538; doi:10.1371/journal.pntd.0009171)

**S3 Figure: Bacterial CFU quantification by selective culture-dependent medium during *Brucella abortus* orally infection.** WT or Nlrp6*^-/-^* mice received a single oral dose of *B. abortus* and 3 days after, microbiota evaluation was assessed in feces. Samples were plated onto Blood agar (Aerobic) (A), Mannitol agar (B), MacConkey agar (C), Brain-Heart infusion agar (D), BBE agar, MRS agar (F) and Blood agar (Anaerobic) (G). Samples were either incubated in aerobic (A-D) or anaerobic (E-G) atmosphere. Statistical analysis was performed by One-way ANOVA followed by Newman-Keuls’ post test. **** indicates p < 0.001


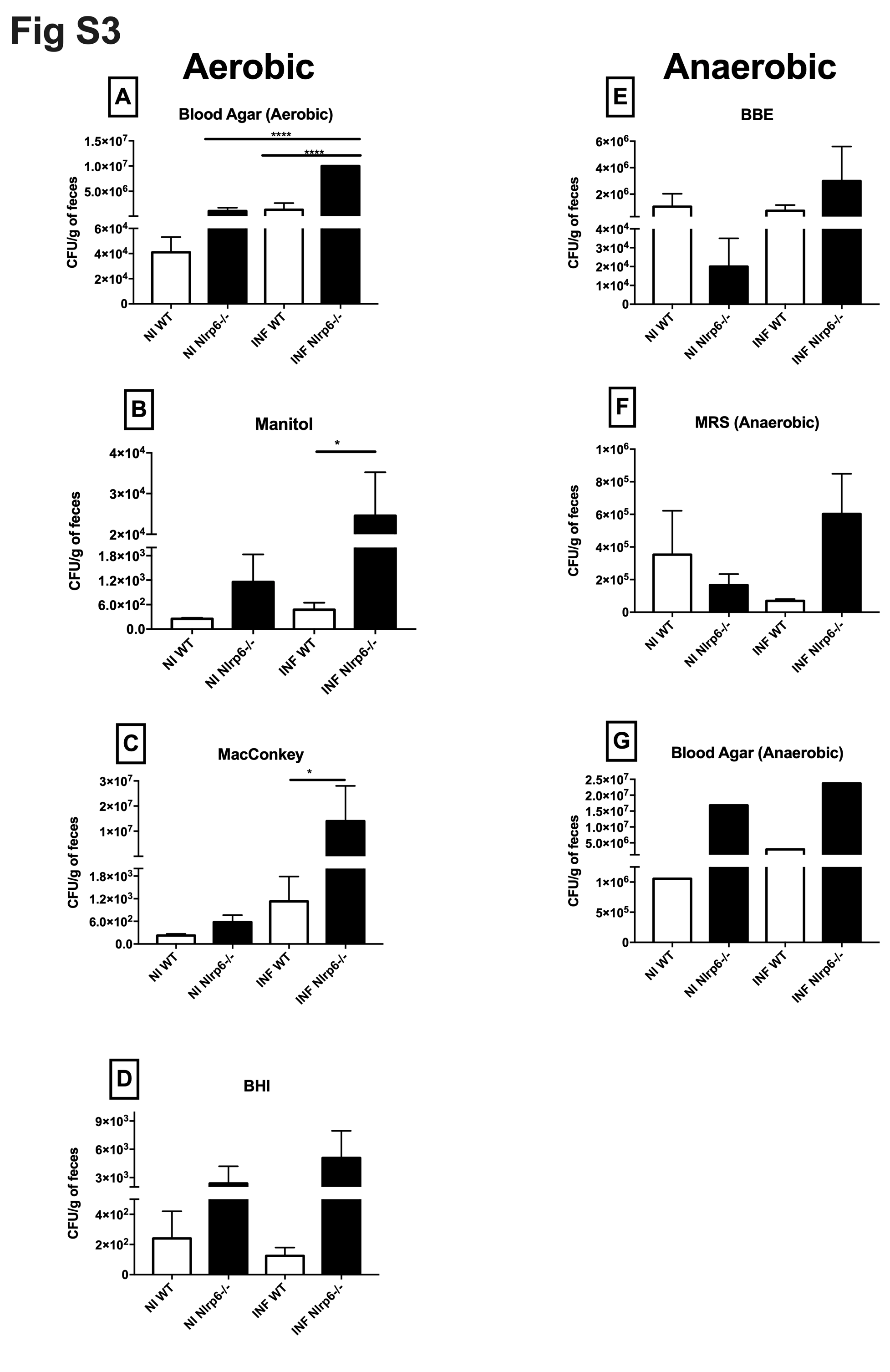

Supplement: S3 Fig — WT or Nlrp6-/- mice received a single oral dose of B. abortus and 3 days after, microbiota evaluation was assessed in feces. Samples were plated onto Blood agar (Aerobic) (A), Mannitol agar (B), MacConkey agar (C), Brain-Heart infusion agar (D), BBE agar, MRS agar (F) and Blood agar (Anaerobic) (G). Samples were either incubated in aerobic (A-D) or anaerobic (E-G) atmosphere. Statistical analysis was performed by One-way ANOVA followed by Newman-Keuls’ post test. **** indicates p < 0.001. (DOCX) [file pntd.0009171.s003.docx]
